# The eyes of the deep diving hooded seal (*Cystophora cristata*) enhance sensitivity to ultraviolet light

**DOI:** 10.1242/bio.011304

**Published:** 2015-05-11

**Authors:** Chris Hogg, Magella Neveu, Lars Folkow, Karl-Arne Stokkan, Jaimie Hoh Kam, Ron H. Douglas, Glen Jeffery

**Affiliations:** 1 University College London, Institute of Ophthalmology, London EC1V 9EL, UK; 2 Moorfields Eye Hospital, London EC1V 2PD, UK; 3 Department of Arctic and Marine Biology, University of Tromsø, 9037 Tromsø, Norway; 4 Department of Optometry and Visual Science, City University London, London EC1V OHB, UK

**Keywords:** Ultraviolet, Retina, Enhanced Contrast, Hooded Seal

## Abstract

The mammalian visual range is approximately 400–700 nm, although recent evidence suggests varying ultraviolet (UV) extensions in diverse terrestrial species. UV sensitivity may have advantages in the dim, blue light shifted environment experienced by submerged marine mammals. It may also be advantageous when seals are on land as UV is reflected by snow and ice but absorbed by fur, enhancing visual contrast. Here we show that the pelagic hooded seal (*Cystophora cristata*) has a highly UV permissive cornea and lens. Seals like other carnivores have a tapetum lucidum (TL) reflecting light back through the retina increasing sensitivity. The TL in this seal is unusual being white and covering almost the entire retina unlike that in other carnivores. Spectral reflectance from its surface selectively increases the relative UV/blue components >10 times than other wavelengths. Retinal architecture is consistent with a high degree of convergence. Enhanced UV from a large TL surface with a high degree of retinal convergence will increase sensitivity at a cost to acuity. UV electrophysiological retina responses were only obtained to dim, rod mediated stimuli, with no evidence of cone input. As physiological measurements of threshold sensitivity are much higher than those for psychophysical detection, these seals are likely to be more UV sensitive than our results imply. Hence, UV reflections from the TL will afford increased sensitivity in dim oceanic environments.

## INTRODUCTION

Hooded seals (*Cystophora cristata*) spend approximately 90% of their time submerged in oceanic waters, with the capacity to dive to >800 m and to remain submerged for almost 1h (Folkow and Blix, 1995). Hence, their visual environment is restricted as at progressive oceanic depths light becomes dimmer and spectrally shifted towards shorter wavelengths. However, significant amounts of UV penetrate several hundred meters and some deep-sea crustaceans on which these animals feed have visual pigments maximally sensitive to UV (Losey et al., 1999; Johnsen, 2004; Vantrepotte and Melin, 2006; Cronin and Frank, 1996).

The mammalian visual range potentially extends from the UV to approximately 700 nm, although the degree of UV sensitivity is variable (Bowmaker, 2008; Douglas and Jeffery, 2014). Many mammals have a single rod with maximum sensitivity around 500 nm and two cone types, maximally sensitive at 415–460 nm and 500–570 nm. However, all marine mammals studied, except manatees, have lost their short wavelength cones leaving a single longer wavelength sensitive cone subtype present at very low density (Bowmaker, 2008; Jacobs 1993; Crognale et al., 1998; Peichl et al., 2001). The reason for this may be that their migration from land to water was in muddy estuaries that absorbed shorter wavelengths. Hence, loss of short wavelength cones would not be disadvantageous (Peichl et al., 2001). This loss may also relate to the high scattering coefficients of short wavelengths in water that may confer disadvantage (Levenson and Dizon, 2003).

Diving mammals may have specific mechanisms to enhance shorter wavelength sensitivity to take advantage of oceanic UV availability in an otherwise dim visual environment. One such mechanism might involve the tapetum lucidum (TL), a reflective layer behind the central retina. The TL normally has chromatic properties and usually occupies 20–30% of the central retinal area over which the retinal pigmented epithelium (RPE) lacks melanin allowing light to pass. In terrestrial carnivores it is commonly golden in reflection (Johnson, 1901; Walls, 1942).

Here we ask if a deep diving seal has the ability to detect UV components present in oceans by measuring the spectral transmission of their optical media and their retinal sensitivity to UV. We aim to demonstrate that the chromatic properties of the TL, that have been largely ignored, may be key in tuning an animal's visual system to a specific light environment.

## RESULTS

### Ocular media let UV light enter the eye and reach the retina

**Fig. 1.**
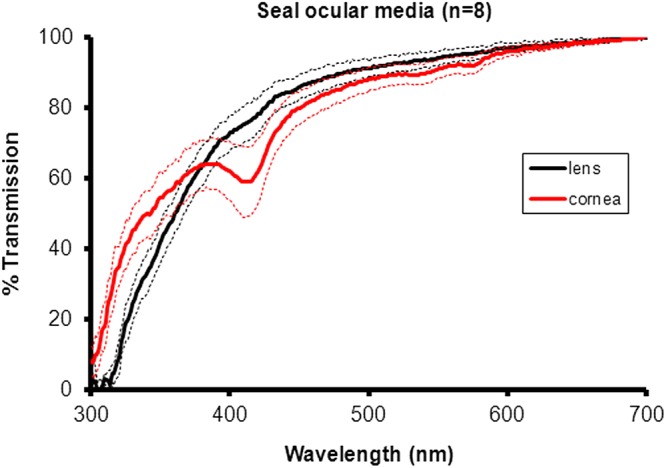
**Average spectral transmission profiles for 8 hooded seal corneas (red) and lenses (black) collected from five animals**. Dotted line represents ±1 S.D. Both corneas and lens transmitted UV down to approximately 300 nm. The dip in corneal transmission at approximately 410 nm was due to small amounts of blood on the cornea that could not be removed

The cornea and lens transmitted 55% and 46% of the UVA respectively at around 350 nm (Fig. 1). Spectral dips, particularly at 410 nm, are due to oxygenated blood that could not be removed from the tissue. This arose during enucleation and adhered while freezing and would not be present *in vivo*.

**Fig. 2.**
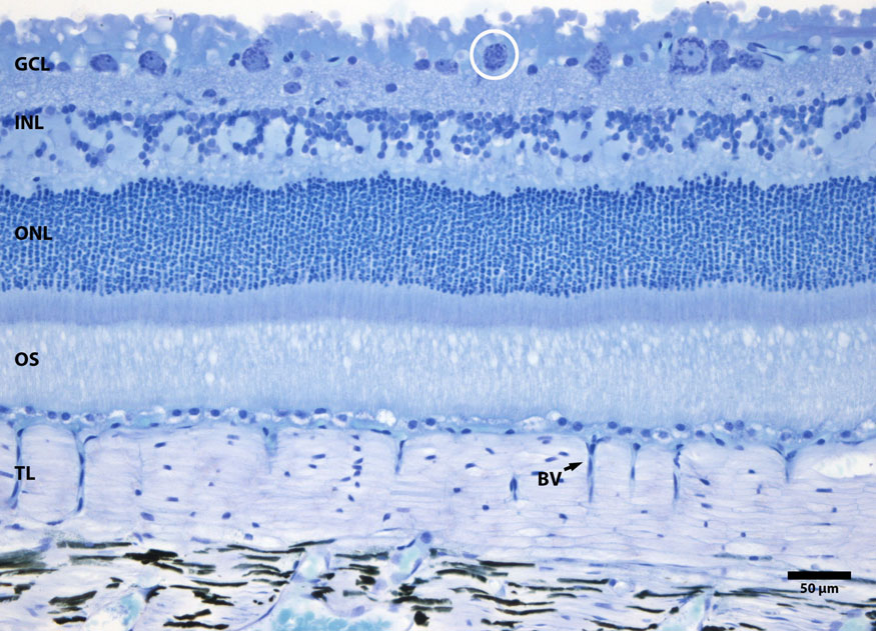
**Histological resin section of the seal retina and TL.** The TL which is sandwiched between the retina and the outer choroid is composed of multiple horizontal layers. The outer nuclear layer (ONL) containing photoreceptor nuclei is regular and relatively normal in terms of the carnivore pattern. However, the inner nuclear layer is very unusual as it has no laminar structure and contains fewer cells than expected. Likewise there are few cells in the ganglion cell layer (GC) and those large enough to be neuronal contain large amounts of Nissl substance, consistent with elevated protein synthesis. The overall architectural pattern in this retina is one with a high degree of convergence. Abbreviations. BV, blood vessel. GL, ganglion cell layer. INL, inner nuclear layer. ONL, outer nuclear layer. OS, outer segments. TL, tapetum lucidum.

The retina has a high pattern of convergence. Histological examination of the TL in section revealed that it was approximately 100 µm wide and only changed marginally in thickness between the centre and the periphery. This is consistent with the TL providing a consistent reflection across a wide region of the retinal surface. Generally the TL was around 30 cuboid cells deep, but there was no indication of the reflective material within them that forms the structural basis of the mirror (Walls, 1942). The thickness of the TL clearly provided a barrier to outer retinal perfusion. While the blood supply to the choroid was extensive, the supply to the outer retina only occurred in blood vessels that projected through the TL in an orthogonal pattern. These branched very tightly under the RPE (Fig. 2).

**Fig. 3.**
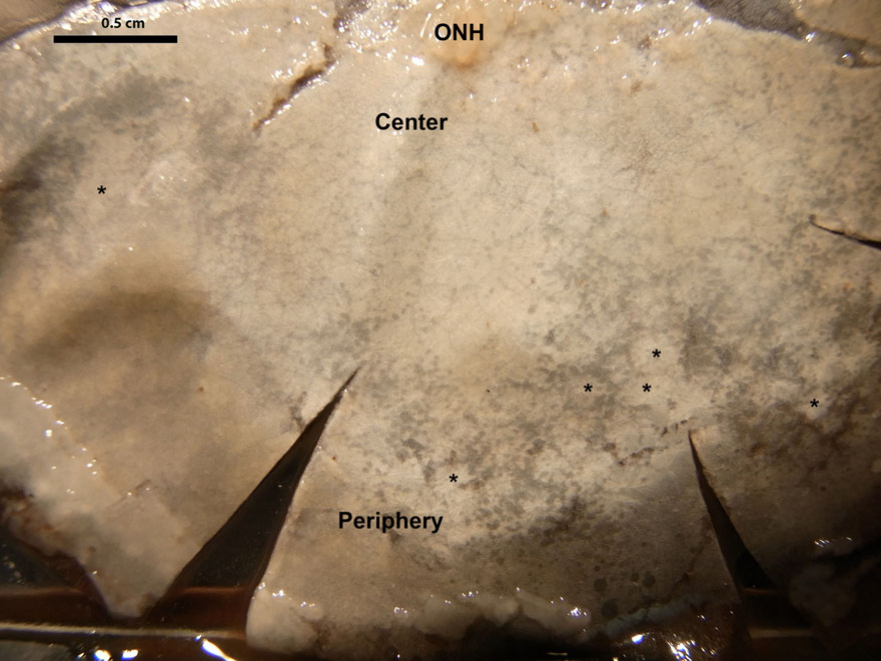
**Low power hemi retinal image of the RPE surface showing the albino central and equatorial retina and pigment distribution in the periphery.** The image corresponds to approximately half the retinal RPE area. The optic nerve head (ONH) is to the top. Peripheral pigmentation is commonly circular around albino patches. Some of the albino parches in the periphery have been marked with a star for identification. Similar faint pigment patterns can be seen more centrally. However, the majority of the retina is albino. Magnification ×1.75.

In the outer retina, the outer nuclear layer (ONL), which contains the photoreceptor nuclei, had a clear laminar structure and was around 100µm thick, contained approximately 11–13 layers of individual cells. This showed limited variation over the retinal surface, consistent with a lack of retinal specialisation. Outer segments were between 50–100 µm in length. However, the inner nuclear layer (INL) was abnormal in comparison to the usual mammalian pattern. There was no clear laminar structure so estimates of its thickness could not be provided. The innermost layer of the INL was the only layer that was relatively continuous, but only at the single cell level. In outer regions of the INL there were large gaps in the distribution of cells that contained no obvious structure or nuclei (Fig. 2). Similarly, ganglion cells were relatively rare. Those present had large amounts of Nissl substance indicative of high levels of protein synthesis (Fig. 3). These architectural patterns are associated with a high degree of retinal convergence with a large photoreceptor population innervating relatively small bipolar and ganglion cell populations. These cellular patterns changed little between centre and periphery and were consistent across separate retinae examined. High patterns of retinal convergence are likely to undermine acuity but will have advantage in a low luminance environment providing greater sensitivity.

### The tapetum lucidum

The whole-mounted low power tissue preparations of the RPE surface revealed unusual patterns of retinal pigmentation. In most mammals that have a TL, retinal pigment is absent from the central retina, revealing the TL without interference from melanin. This albino region is relatively small commonly account for around 25–35% of the retinal area (Walls, 1942; Johnson, 1901). In the seals examined here more that 90% of the retina was albino. Pigment was absent from the central retina apart from a few single cells that were in roughly circular patterns. But melanin density in these RPE cells was very low. Similar patterns extended into the equatorial region (Fig. 3). In the periphery, pigmentation was more marked but still only a minority of cells contained melanin. Those cells that were pigmented appeared to have less melanin than commonly found in mammalian RPE but more than at central locations. This was reflected in their grey rather than black colour. RPE cells were large being approximately 30–40 µm in diameter. Again, peripheral albino regions were often circular surrounded by a thin line of pigmented cells (Fig. 3). When the number of cells containing pigment were counted at central and peripheral locations in two eyes means of approximately 15 were found centrally, while means of approximately 145 were found in the periphery within a region of 0.04 cm^2^, giving a ratio of approximately 1:10 between the two retinal regions. It was not possible to estimate the percentage of pigmented cells at defined locations as it was not possible to identify the number of albino cells or their size.

### Tapetal reflections enhance UV

**Fig. 4.**
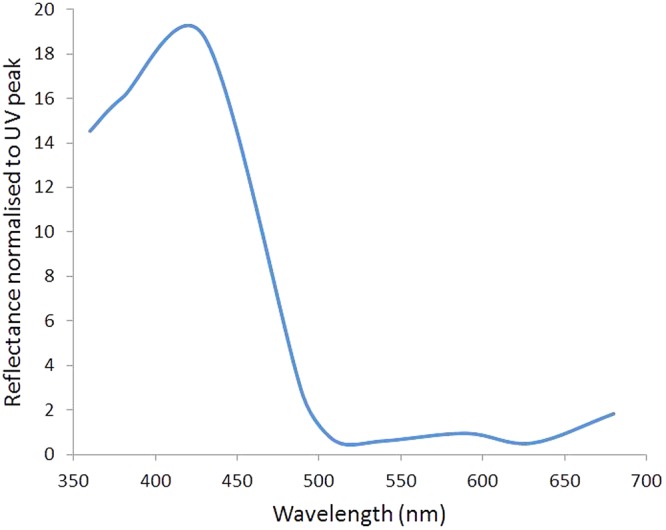
**The relative reflectance of the seal TL from 360 nm–680 nm.** The overall responses have been averaged to provide a smooth function. Relative reflections are much greater at the shorter wavelengths. Hence the TL increases the relative reflection of short wavelengths. Data from two animals was very similar. In spite of the small amounts of pigment in the peripheral retina there was no obvious difference in the patterns of reflection from central and peripheral regions. The Y axis is a relative measure of the intensity of the reflection.

Reflectance measurements made using the integrating sphere containing a spectrum of LEDs (360 nm–670 nm) gave clear consistent patterns that varied little with eccentricity, consistent with the widespread albino pattern of retinal pigmentation. In each case there was a peak reflection recorded in association with a stimulating LEDs spectral output, but these relative reflections varied in their magnitude. Those above 510 nm were of roughly similar magnitudes with the 475 and 670 nm giving the larger reflections compared to 510, 590, 630 nm. But all of these were much smaller than the reflections to shorter wavelengths in association with stimulation at 360, 375 and 425 nm, where the relative increase of the reflectance was >10 times greater than at any of the longer wavelengths. Hence, the reflection from the TL differentially reflected the shorter wavelengths including the UV preferentially compared to the longer wavelengths (Fig. 4).

**Fig. 5.**
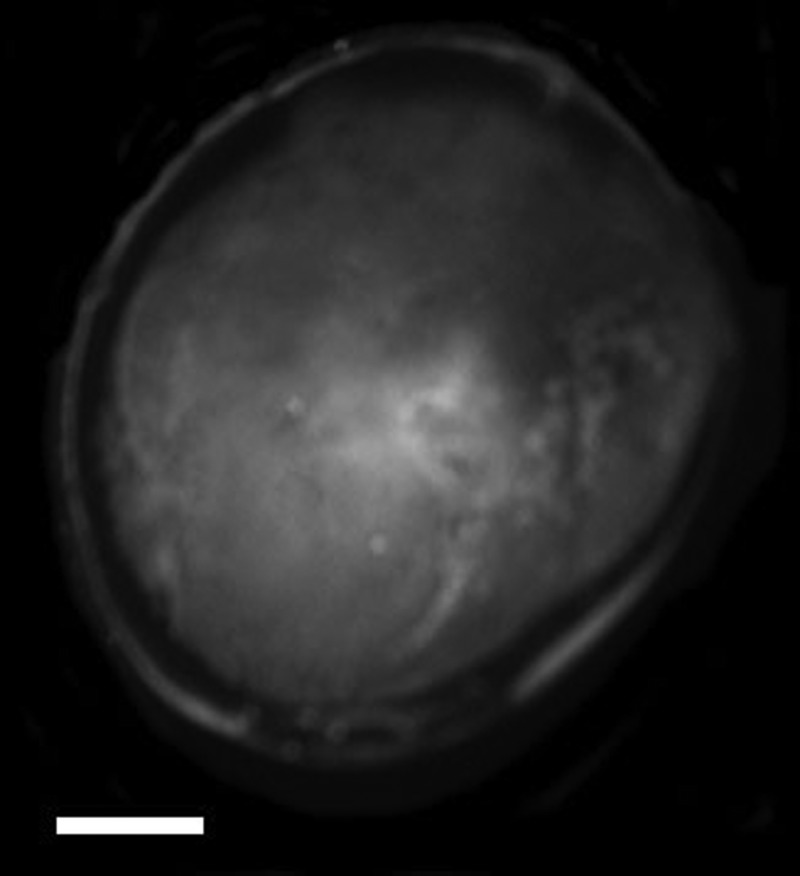
**UV reflections from the eye cup.** To confirm that there was a UV reflectance from the TL, an eye cup with the cornea, lens and retina removed was imaged in the dark with a UV sensitive camera with a filter over the lens blocking light above 380 nm. This was illuminated with UV between 360 nm and 380 nm. A clear reflection can be seen from the inside of the eye cup. The brightest central spot is due to direction of reflection. The diameter of the eye cup opening is approximately 6 cm. Scale bar=1 cm.

In confirmation of the spectral measurements, UV photography in the absence of visible light showed clear UV reflections from the eye cup (Fig. 5). Here the eye cup was illuminated with 360 and 380 nm LEDs. As the camera attenuated 50% of the UV signal and had low resolution in the UV range, the image is a conservative representation of the degree of UV reflectance.

### The retina responds physiologically to UV

**Fig. 6.**
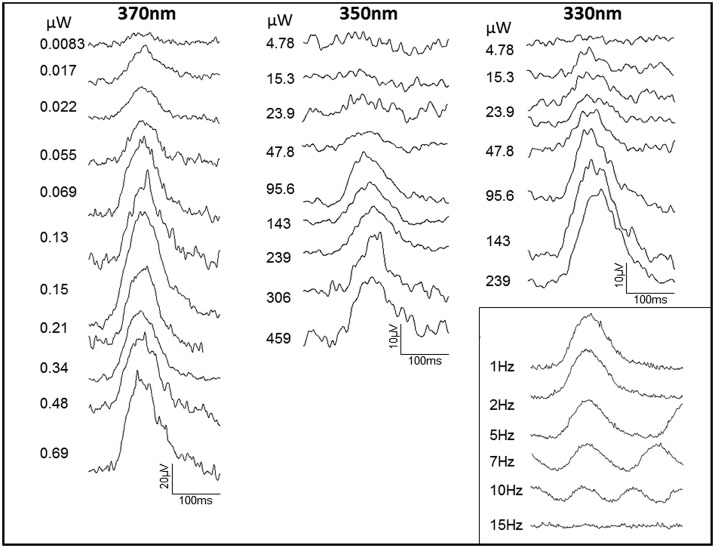
**ERGs recorded to UV stimuli at 330 nm, 350 nm and 370 nm to increasing (top to bottom) stimulus intensity.** ERGs at 330 nm and 350 nm are similar in amplitude. ERGs at 370 nm require less energy to elicit a response of similar amplitude. ERGs at 370 nm are double the amplitude of ERGs at 330 nm and 350 nm. Data are representative for all animals. Insert**:** ERGs to UV stimuli (370 nm) at increasing temporal frequencies (top to bottom). Responses are clear from 1 to 10 Hz but undetectable at 15 Hz. Similar data were obtained from two animals. Rod mediated responses do not extend beyond 15-18 Hz (Hecht and Shlaer, 1936: Kelly, 1974). Hence, responses here were limited to rod function.

ERG UV responses were present and similar in all animals. At low luminance the first response detected was the prominent positive b-wave and with increasing UV stimulus intensity the b-wave amplitude increased, seen running top to bottom in Fig. 6. ERGs recorded to the three stimulus wavelengths show that less energy was required to elicit a response at 370 nm compared the other UV wavelengths (Fig. 6). All responses reported are within the rod mediated intensity range.

At low luminance levels, only rods contribute to the ERG (Rushton and Powell, 1972; Nordby et al., 1984). With increasing stimulus intensity rod and cone responses interact, with increasing contributions from cones, although cone density in the seal is very low and confined only to L/M cones that are spectrally distance from the UV (Peichl et al., 2001). However, rods and cones have different temporal response characteristics, with rods not responding above 15–18 Hz (Hecht and Shlaer, 1936). Hence, 370 nm stimuli were used at increasing temporal frequencies to reveal if b-wave responses were rod or cone driven. Clear responses were obtained between 1 Hz and 10 Hz, but were not present at 15 Hz (Fig. 6 insert). Hence, there is no evidence that cones mediate the UV response. Cone responses could, however, be recorded in response to white light at frequencies >15 Hz used to established the ERG profile in each animal (not shown).

## DISCUSSION

This study reveals that UV passes through the hooded seal ocular media, and that there is a relative increase in the reflection of shorter wavelengths by the TL compared to those that are longer by more than a factor of 10. Further, the seal retina responds to this light physiologically. UV light sensitivity was higher at longer UV wavelengths, where more UV enters the eye. Specific UV sensitive cone opsins have been documented in some small mammals (Bowmaker, 2008). UV sensitivity is likely to be a feature to a greater or less extent in a wide range of mammals that have ocular media permissive for it, but where there is no evidence of a UV specific opsin (Douglas and Jeffery, 2014; Stokkan et al., 2013). In such animals UV may drive both photoreceptor types (Hogg et al., 2011). However, cones are very rare in seals and short wavelength cones are missing (Peichl et al., 2001). Hence, our UV responses in this animal were most likely rod mediated.

The hooded seal lens is highly UV permissive. Rodent lenses that are much smaller let through more UV. A mouse lens (diameter 3.85 mm) transmits 81% of UVA (Douglas and Jeffery, 2014; unpublished results). Such a lens scaled up to that of the seal would transmit 46% of UVA, which equals the amount transmitted in the seal. Hence, per mm path length the seal lens is as transparent as that in mouse, which has a UV sensitive cone opsin. Interestingly, the reindeer, whose retina responds to UV (Hogg et al., 2011), has a lens that lets through less UVA (27%) than the seal lens, although it is significantly thinner (means 10 mm vs 17 mm).

The mammalian visual range was thought to be limited to approximately 400–700 nm in most species and to only extend into shorter wavelengths in a few smaller mammals that had UV sensitive opsins with peak sensitivities around 370 nm (Bowmaker, 2008). But recently it has been shown that the ocular media of many mammals is to a greater or lesser extent UV permissive (Douglas and Jeffery, 2014). However, although Douglas and Jeffery (Douglas and Jeffery, 2014) examined 38 mammalian species including carnivores, aquatic mammals were not included in the analysis of both the cornea and lens. Even mammals lacking a specific UV opsin will be UV sensitive if the ocular media allow it to pass, as all visual pigments absorb significant amounts of UV if the energy level is sufficient. Hence, extension of the visual range into the shorter range will increase sensitivity to available light. In the seal this may be particularly significant due to its reduced light environment.

### The tapetum and seal vision

Our data show that the seal TL is effective at magnifying the relative UV component of incoming light. Measurements of reflected light from whole mount preparations showed that UV reflection was >10 times greater than any other part of the spectrum apart from at 425 nm. These data are supported by eye cup imaging with a UV stimulus and a UV sensitive camera.

The TL varies considerably between mammalian species, having different structural forms and reflective material, consistent with it being an example of convergent evolution (Walls, 1942). In the seal the TL is cellulosum, which is formed of layers of cells containing refractive material. The refractive material varies between different mammals, but here is likely formed of zinc-cysteine crystals common to carnivores. (Duke-Elder, 1958; Pedler, 1963; Lesiuk and Braekevelt, 1983). But in other respects the seal TL is markedly different from that in other carnivores, particularly in its large size and lack of any colouration. Most carnivores, have a golden TL located in a defined region in the dorsal/temporal retina corresponding to the area centralis (Johnson, 1901; Walls, 1942). The striking feature in the seal is the almost complete absence of retinal melanin in most RPE cells. It is only found to any significant degree in a limited population of cells in the periphery where melanin density remains very low. We estimate that about 90% of the seal retina is albino. To match this, the TL did not thin noticeably towards the periphery resulting in an almost continuous pan retinal white reflective surface.

In only two other species do we find a similar pattern of tapetal pigmentation and both are deep diving marine mammals, implying that this unusual feature affords advantage in deep oceanic environments. Johnson (Johnson, 1901) published a comprehensive study of the TL in a large range of mammals. He describes the fundus of the sperm whale as “mottled with patches of dark grey”. Unfortunately, no other details are provided. How Johnson was able to image the fundus of this whale is not explained as the animals optics would cloud soon after death and it is unlikely that he imaged the eye while it was alive. Had he done so, this must have been with an ophthalmoscope as with all the other animals he studied, and hence his description must only be for the central retina. In contrast, ours analysis is for the complete retina. A comment regarding the absence of pigment over a large retinal area was also made for another deep diving seal, the Antarctic Weddell seal (Welsch et al., 2001). But the TL was not the primary interest of this study and there is no more than a brief comment and a single image taken in transverse section at low power.

The absence of pigment over such a large area is likely to increase sensitivity due to a larger mirror for a second light pass, but at the price of reduced acuity as this will be associated with increased light scatter, particularly for the shorter wavelengths. This play off is reflected in the organisation of the retina. The seal retina has a significant photoreceptor population, but an impoverished INL and ganglion cell layer, consistent with a high degree of retinal convergence. This also appears to be the case for two other deep divers, the grey whale (Mass and Supin, 2007) and the Weddell seal (Welsch et al., 2001) who appear to have a similar INL. However, our statement in this respect is based on images in these publications rather than analysis of such features by the authors. But it does imply that two separate groups of aquatic deep diving mammals, a cetacean and a carnivore, have both made significant changes to their retinae, specifically reducing their inter neuron populations to increase convergence. Given that the TL covers such a large area and that it preferentially reflects shorter wavelengths that will have greater scattering coefficients, it is not surprising that the animal's retina has evolved to maximise signal detection at the expense of acuity. Hence, the organisation of the TL and the architecture of the retina appear to have evolved to resolve a similar problem associated with limited light that is spectrally shifted.

While the seals eye appears to have undergone a specific evolutionary path, there is significant evidence that vision remains important to this animal while diving. Its eye is extremely well vascularised with counter-current vascular arrangements that are probably important in preventing excess heat loss while diving in cold waters (Folkow et al., 1988). Retinal perfusion in seals subjected to simulated diving in the laboratory declines only marginally compared with reductions of 80–90% for many other parts of the body (Zapol et al., 1979). Blix et al. (Blix et al., 1983) report that “the retina of the eye received the same perfusion after 10 min submersion (120 ml/min 100 g) as before the dive”. Further, while dark adaptation times in shallow diving seals are significantly better than in man being approximately 20 min to reach maximum sensitivity, those of a deep diving elephant seal were only 6 min (Levenson and Schusterman, 1999). This is approximately the time taken for this animal to dive 700 m, which is a common foraging depth. This ability is likely due to the animal having a large highly contractile pupil. When fully dilated it will maximise sensitivity, but on the surface may contract to such an extent that rods may not fully bleach. (Le Bouef and Laws, 1994). Hence, retinal function is likely to be of importance during diving.

### Retinal recordings

The data presented for the differential reflectance of the TL are consistent with that from our physiological recordings. The only other large mammal in which UV responses have been recorded is the reindeer. Here both rods and cones respond to these short wavelengths (Hogg et al., 2011). Presumably at higher luminance this occurs via short wavelength cones, whose peak sensitivity is closest to UV at around 437 nm. But such short wavelength cones are absent in seals, including the hooded seal studied here (Peichl et al., 2001). Visual pigments are described in habor seals (*Phoca vitulina*), where the single cone peak sensitivity is 510 nm (Crognale et al., 1998). If hooded seals are similar, such cones are unlikely to be UV responsive at the intensities used here as they are too long wavelength shifted, although we could stimulate them with white light (data not shown). Low cone density may also contribute to the reason why Levenson et al. (Levenson et al., 2006) failed to obtain cone responses in pinnipeds using ERG recordings. Consequently, hooded seals probably only respond to UV at low luminance where rods function. As they are at sea >90% of the time, and approximately 90% of this submerged (Folkow and Blix, 1995), these animals will be partially dark adapted most of the time and their small cone populations will be insensitive. This would be particularly true in winter.

### The oceanic light environment and seal vision

The optics of oceanic light are consistent with our ERG results. Oceanic sunlight is attenuated with depth, although enough reaches about 1000 m in clear water to allow vision in the most sensitive animals (Denton, 1990). The spectrum also becomes restricted towards the short wavelengths (Johnsen, 2004; Vantrepotte and Melin, 2006). Enough UV reaches depths of several hundred meters to allow its perception in some crustaceans with visual pigments maximally sensitive to it (Cronin and Frank, 1996). But at such depths light in oceanic environments has another source in the form of bioluminescence (Turner et al., 2009). It is suggested that southern elephant seals (*Mirounga leonina*), uses this in prey localisation (Vacquié-Garcia et al., 2012). However, UV is not produced by bioluminescence (Herring, 1983; Johnsen et al., 2012) and as such is unlikely to be relevant to our study.

Enhanced UV sensitivity may also play a role above the surface. UV imaging has been used in seal aerial censuses (Lavigne and Øritsland, 1974) because UV is absorbed by their fur (Reynolds and Lavigne, 1981). Consequently, it enhances contrast against the UV reflecting background of snow/ice, and for seals above the surface, this increases their ability to avoid polar bear (*Ursus maritimus*) predation as the bears white fur will absorb UV against a UV reflective background from snow and ice. For the same reason it will facilitate detection of conspecifics. This may be important when young are born in March when the sun remains low on the horizon, causing relatively high UV levels due to atmospheric Rayleigh scatter.

We have shown that the anterior segment of hooded seal eyes are highly UV permissive and that their TL magnifies this spectral component, increasing sensitivity at the expense of acuity. The animals' retina appears to have evolved along similar lines and these different factors contribute to retinal UV sensitivity. However, we believe that seals may be much more UV sensitive than our data suggest as sensitivity thresholds measured with psychophysical methods are log units lower than those measured with physiological techniques similar to those employed here (Ruseckaite et al., 2011).

## MATERIAL AND METHODS

### Animals

Twelve 2–3 year old hooded seals (*Cystophora cristata*) weighing 84–94 kg were used. These were caught as weaned pups in the Greenland Sea pack ice (permits from The Royal Norwegian Ministry of Fisheries and The Royal Danish Ministry of Foreign Affairs) and kept at The University of Tromsø in large indoor sea water pools under light/dark cycles mimicking natural light conditions. Experiments were undertaken under Norwegian Animal Research Authority permit (no 3523). Animals were primarily used for experiments other than those reported here and tissues were harvested from them at the termination of these. A series of experiments were undertaken primarily focused on the potential UV abilities of this animal's visual system. The spectral transmission of 8 corneas and lenses were examined from 5 adult animals of both sexes from the same stock. Six animals were used to examine the TL and retina histologically in section and in whole mount. These included optical experiments to examine the TL spectral reflection to wavelengths between 360–670 nm. An additional 5 animals were used in electrophysiology experiments where the electroretinogram was recorded to UV light.

### Measurements of lens and corneal spectral transmission

Eyes (∼5 cm diameter) were enucleated at death and immediately frozen. Subsequently they were thawed and the corneas and lenses removed and washed in PBS. The lens equatorial diameter was approximately 20 mm and the axial thickness about 17 mm. Lenses and corneas were air mounted in holders constructed of aluminium and containing a hole snuggly fitting either the lens or cornea. This was placed in a standard quartz glass cuvette so that the tissue was in the middle of the measuring beam and scanned in 1 nm steps between 300–700 nm with a Shimadzu UV-2101PC spectrophotometer fitted with an integrating sphere. Control experiments with mammals and fish showed no effect of freezing on lens transmission (Douglas and Jeffery, 2014). Transmission at 700 nm was set at 100%.

### Histology of the TL and retina

Eyes were enucleated at death and fixed in 10% formalin. Subsequently, the anterior chamber and lens were removed and the retina, RPE and choroid dissected free from the sclera in 4 eyes. This resulted in a whole mount preparation approximately 6.5 cm in diameter. From this whole mount 9 regions were removed from central, equatorial and peripheral retina that were approximately 0.5–0.7 cm^2^. These were dehydrated through a graded series of alcohols between two microscope slides, embedded in resin (Technovit 7100, TAAB, UK), sectioned at 5 µm, mounted onto slides, stained with toluidine blue and cover slipped. Regions from identified locations were also examined in from the RPE sheet. Here the tissue was examined at 400× magnification while wet and the number of cells containing pigment in the RPE were counted for central and peripheral regions. A total of 800 cells were examined in two preparations from two different animals within regions measuring 0.04 cm^2^.

### Analysis of the spectral reflection of the TL

Eyes from two seals where enucleated and fixed in 10% formalin as above. The anterior chamber and lens were removed and the retina dissected free leaving the RPE surface exposed. The RPE and attached choroid were removed, washed in phosphate buffer and photographed in whole mount at low power providing images of patterns of pigmentation. These preparations were then flat mounted in a large petri dish and kept moist. To examine the TL reflection across the spectral range of interest (350–700 nm), a light source was constructed using LEDs embedded into a table tennis ball whose interior was coated in spectrally neutral white paint. The energy to each LED was matched, providing similar output power within an integrating sphere at the following nominal wavelengths; 360, 375, 425, 475, 510, 590, 630 and 670 nm. The mean half power band width of the LEDs used was typically 20 nm. We adopted this method of wavelength stimulation to examine the TL reflectance because the animals oceanic light environment is unlikely to be represented by a normal white light source as it spends ∼90% of its time under the water. The internal reflections within the integrating sphere from the LEDs were collected via a fibre optic that could transmit them to the surface of the TL with an illuminated area of approximately 5 mm^2^ at a distance from the tissue surface of approximately 1.5 mm. The fibre optic cable had a coaxial outer sleeve that collected the reflected light from the TL and fed it into an Ocean Optics spectrometer (USB2000+UV-VIS-ES) with a sensitivity range from 200–850 nm. Hence, the reflected light from the TL surface could be analysed in terms of its spectral components and their magnitude in relation to the stimulating wavelengths of the LEDs. The output of the spectrometer was fed into a PC that displayed the wavelength along the X axis and the magnitude of the reflected light along the Y axis and hence a relative spectral reflection of the TL. Measurements of these reflections were made in strips running out from the centre of the retina with an X-Y micromanipulator in steps. These have been presented as an average as there was little variation irrespective of retinal location. However, it was not possible to take an average across retinae because while the profiles from different eyes were the same, their overall magnitude differed markedly. Hence, it would not have been possible to pool the data retaining a common Y axis. Approximately 10 strips were analysed in two preparations from two different eyes from separate animals. These ran from central to peripheral retina using the optic nerve head as a landmark.

An additional method was used to examine potential UV components of the TL reflection by imaging the total TL surface in situ in one cup under UV illumination. The eye cup with the cornea, lens and retina removed was washed in phosphate buffer and exposed to UV light in a dark room from a source containing UV LEDs at 360, 375 and 380 nm. The total irradiating energy was 0.0005 w/cm^2^ at a distance of about 20 cm. This was imaged with a UV sensitive monochrome camera (Watec WAT-902h Ultimate) with an uncoated 25 mm f1.9 Cosmicar lens. A Hoya U340 UV filter was placed over the camera lens blocking light above 380 nm. The camera had been pre-calibrated against the UV LEDs prior to imaging the eye cup. The camera was sensitive down to approximately 350 nm but attenuated UV by approximately 50%. Hence, imaging in the UV provided conservative estimates of the UV reflectance. This camera and filter arrangement had been used previously for UV imaging in the field (Tyler et al., 2014).

### Electrophysiology

Seals were sedated with an intramuscular injection of Zoletil (1.2 mg/kg Forte Vet zolazepam/tiletamine; 100 mg/ml, Virbac, Carros Cedex, France) and a central venous catheter inserted into the extradural intravertebral vein, allowing supplementary sedation (0.2–0.3 mg/kg). This was also used to inject Propofol (0.5–1.0 mg/kg Propofol-Lipuro; 10 mg/ml, Braun Melsungen AG, Melsungen, Germany) to achieve anaesthesia allowing tracheal intubation and manual ventilation with isofluran (Forene, Abbott Scandinavia AB, Solna, Sweden) in air (0.75–1.5%) to maintain anaesthesia. Rectal temperature, heart rate and arterial oxygen saturation were monitored. Seals were warmed with a water-circulated heating table (40°C). After ERG testing, seals quickly recovered (within 10–15 min) and were observed for 2 h before returning to water.

Methods for ERG recordings were similar to Hogg et al. (Hogg et al., 2011) in reindeer where UV responses were explored in another large mammal lacking a UV opsin. The characteristics of the ERG examined are similar across most mammals that have been studied and these have also been undertaken previously in seals (Crognale et al., 1998). Anaesthetised animals were dark-adapted for >30 min. The left eye was dilated (tropicamide 1%, phenylephrine 2.5%) and stabilized with scleral sutures and a gold foil corneal ERG electrode placed under the lid. LEDs (LUX EON. Phillips UK), were placed over the eye that were built into a tube with an internal reflective surface. ERGs were recorded to white light (420–620 nm) to establish baseline responses confirming the presence of the main waves and their relative timing. UV (372 nm, 350 nm, 330 nm) stimuli were then delivered from a diode array at the rear of a 50 mm diameter tube internally coated with a diffuse reflector. UV responses were recorded over energies from 1×10^−6^ µW/cm^2^ to 46 µW/cm^2^. Recordings were made sub threshold, and at increasing intensities to establish threshold responses. Amplitudes and peak times of a-wave and b-waves were measured. The a-wave is generated by photoreceptors and the b-wave by post-receptoral cells. To determine if UV responses were rod or cone driven their temporal characteristics were investigated using stimuli presented at progressive frequencies (Hogg et al., 2011) at a stimulus intensity of 1.2 µW/cm^2^, within the range of overlapping rod and cone function. Rods are unable to following flickering stimuli above approximately 15–18 Hz, while cones are able to follow flicker at higher levels. Hence, this is a method of discriminating the photoreceptor origin of a response.
